# Supplementary material for: MicroRNA-769-3p Acts as a Prognostic Factor in Oral Squamous Cell Cancer by Modulating Stromal Genes
Source: Cancers (Basel). 2022 Sep 8;14(18):4373. doi: 10.3390/cancers14184373 (PMC9496693; doi:10.3390/cancers14184373)
Supplement: Supplementary file 1 [file cancers-14-04373-s001.zip › cancers-1747422-supplementary.pdf]

cancers
MDPI

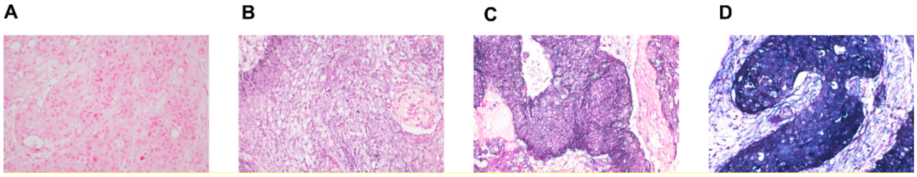

Supplementary Figure S1. ISH analyses of miR-769-3p (×200). (**A**) no staining, (**B**) mild tumor cell staining (grade 1), (**C**) moderate tumor cell staining (grade 2), and (**D**) strong tumor cell staining (grade 3).

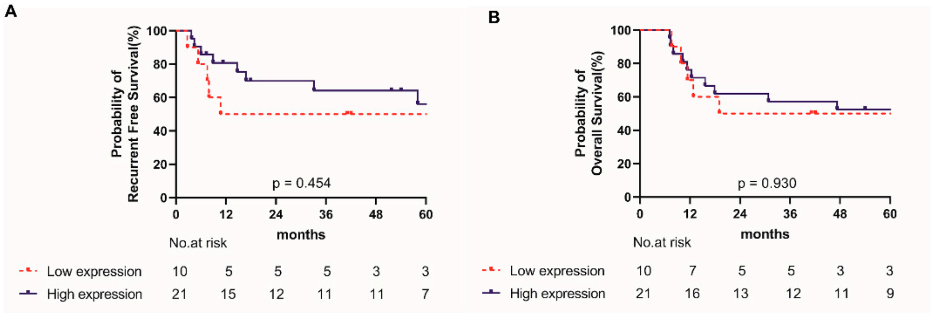

Supplementary Figure S2. Kaplan–Meier survival curves for relapse-free survival (RFS) and overall survival (OS) based on stroma cell miR-769-3p expression in 31 FFPE HNSCC tumor samples. The expression of miR-769-3p expression in stroma showed no significant correlations with RFS (A) and OS (B) (P = 0.454 and P = 0.930, respectively).

Supplementary Table S1. Risk factors for overall survival in 53 patients with OSCC by multivariate analysis.

		Multivariate	
**Variable**		**HR (95% CI)**	***p*-Value**
Age	≥60 vs <60	0.857 (0.366–2.006)	0.722
Sex	Male vs Female	1.526 (0.497–4.684)	0.461
Smoking	Yes vs No	2.005 (0.674–5.960)	0.211
Ethanol *	Heavy vs Mild	1.096 (0.381–3.157)	0.865
Grade	2-3 vs 1	0.399 (0.120–1.333)	0.136
TNM Stage	IV vs I–III	2.843 (1.041–7.760)	0.041
HPV16	Yes vs No	0.227 (0.023-2.199)	0.200
miR-769-3p	High vs Low	0.417 (0.156–1.113)	0.080

OSCC oral squamous cell carcinoma, HR Hazard ratio, CI Confidence interval. * Heavy drinker, heavy use currently: >3 drinks per day.
