# Supplementary figures and images for: MicroRNA-769-3p Acts as a Prognostic Factor in Oral Squamous Cell Cancer by Modulating Stromal Genes
Source: Cancers (Basel). 2022 Sep 8;14(18):4373. doi: 10.3390/cancers14184373 (PMC9496693; doi:10.3390/cancers14184373)

Original blot of Figure 3B.

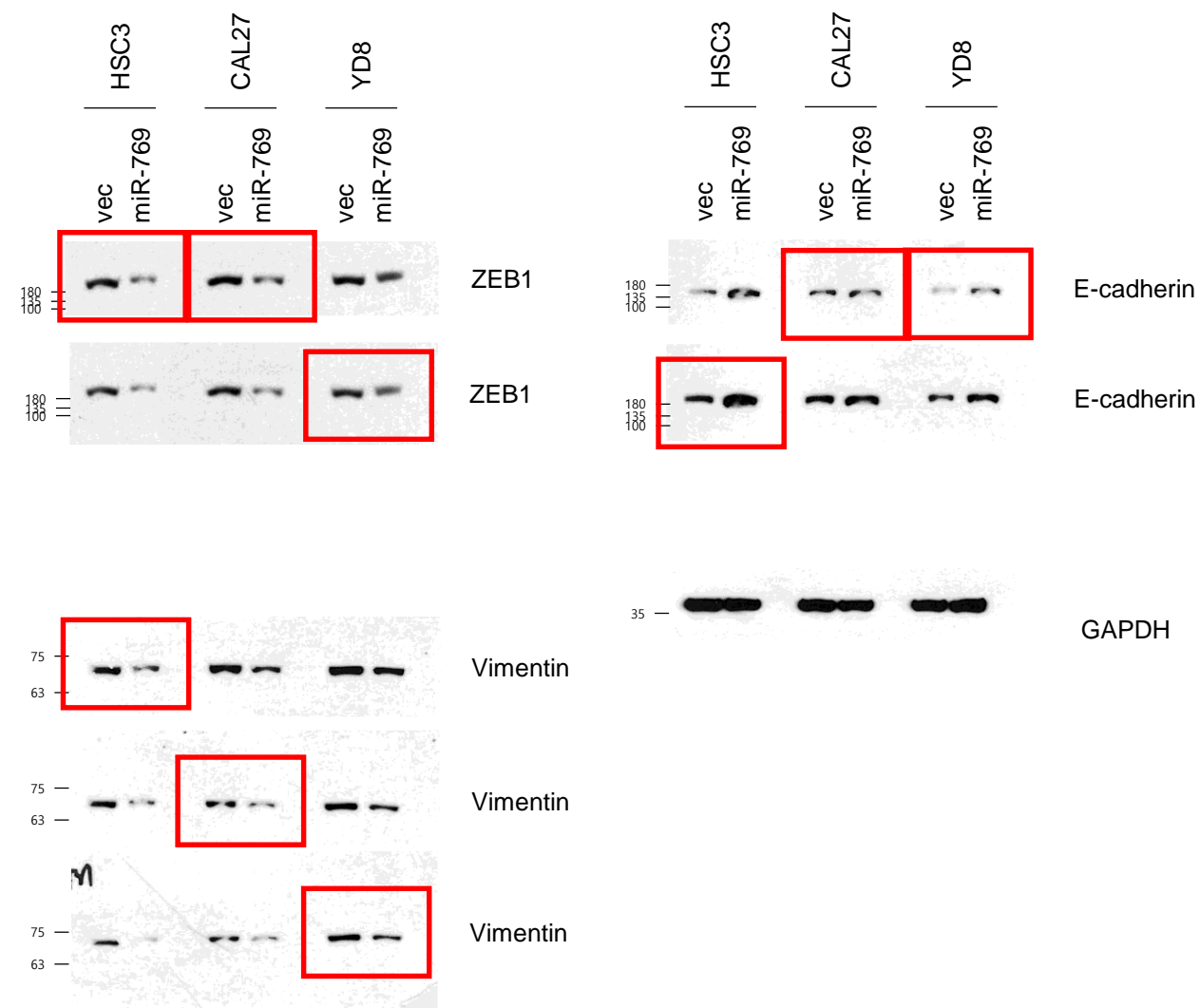

Original blot of Fig 4B.

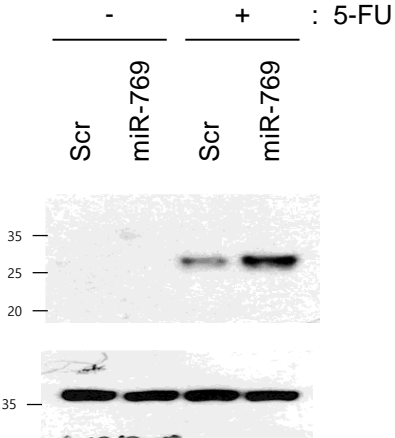

Supplement: Supplementary file 1 [file cancers-14-04373-s001.zip › cancers-1747422-Supplementary File S1.pdf]
